# An Iterative, Participatory Approach to Developing a Neighborhood-Level Indicator System of Health and Wellbeing

**DOI:** 10.3390/ijerph20021456

**Published:** 2023-01-13

**Authors:** Hannah Röhrbein, Jennifer Hilger-Kolb, Kathrin Heinrich, Holger Kairies, Kristina Hoffmann

**Affiliations:** 1 Center for Preventive Medicine and Digital Health (CPD), Medical Faculty Mannheim, Heidelberg University, 68167 Mannheim, Germany; 2 Youth Welfare Office and Public Health Department, Division for Youth Welfare Planning and Public Health Planning, 68161 Mannheim, Germany

**Keywords:** urban health indicators, participatory research, neighborhood, health, wellbeing

## Abstract

Despite increased awareness of the essential role of neighborhood characteristics for residents’ health and wellbeing, the development of neighborhood-level indicator systems has received relatively little attention to date. To address this gap, we describe the participatory development process of a small-area indicator system that includes information on local health needs in a pilot neighborhood in the German city of Mannheim. To identify relevant indicators, we partnered with representatives of the city’s public health department and used an iterative approach that included multiple Plan-Do-Check-Act cycles with ongoing feedback from local key stakeholders. The described process resulted in a web-based indicator system with a total of 86 indicators. Additionally, 123 indicators were perceived as relevant by stakeholders but could not be included due to data unavailability. Overall, stakeholders evaluated the participatory approach as useful. Even though the onset of the COVID-19 pandemic and the lack of some data elements hindered instrument development, close collaboration with public health partners facilitated the process. To identify and target sub-national health inequalities, we encourage local public health stakeholders to develop meaningful and useful neighborhood-level indicator systems, building on our experiences from the applied development process and considering identified barriers and facilitators.

## 1. Introduction

From a socio-ecological perspective, health and wellbeing are not only determined by individual factors (e.g., age or gender) but also by the physical, social and economic environment in which people live [1,2]. As about 55% of the global population lives in urban areas [3,4], living environments play an essential role in shaping individual health status and resources for maintaining health [5]. Neighborhood characteristics in particular have been linked to health and wellbeing through associations with the availability and accessibility of health-care services, opportunities for physical activity and recreation (e.g., parks and green spaces) and healthy, affordable food options [6]. The importance of other neighborhood-level factors such as traffic density, walkability and the existence of social networks to health has also been well documented [7,8].

Differences in these environmental determinants of health, especially when several health risk factors cluster, are likely leading to neighborhood deprivation in underserved areas, thereby contributing to associated health inequalities at a sub-national level [9,10].

Recognizing the essential role of characteristics of urban areas and neighborhoods for health and wellbeing, various global initiatives such as the United Nations’ Sustainable Development Goals (SDGs) and the World Health Organization’s (WHO) Healthy City Networks have pointed to the importance of establishing urban health indicator systems [3,7]. According to Pineo et al., these systems have been defined as “collections of summary measures about the urban environment’s contribution to human health and wellbeing, with a broad interpretation of health that expands to related concepts of quality of life, livability and wellbeing” [11] (p. 419). Such indicator systems are important for local decision-makers in setting priorities, allocating resources, and planning and evaluating area-level health promotion projects [8]. However, data are frequently available at national, regional or city level only, and the development of a comprehensive system of neighborhood-level indicators has received relatively little attention to date [3].

Although neighborhood indicators have been investigated in the past, these often tend to be studied in isolation, for example, when studies investigate single indicators to answer specific research questions [12,13,14]. The isolated consideration of single indicators, without considering the importance of other neighborhood indicators and their potential interactions, may lead to policy or intervention development with a low meaning for the neighborhood context. Moreover, when based on these single indicators or on city-level or less granular data, policies and interventions may contribute to growing inequalities by disregarding local population needs and access barriers to support services. Overall, indicators prominent in one neighborhood may not reflect population needs in other neighborhoods, raising the need to create a granular system of small-scale estimators in which indicators can be mutually studied to reflect the local context in health policy and planning [7,15,16].

Previous research suggests that the acceptability and continued use of indicator systems specifically developed for smaller areas may increase through a clear focus on local needs [7,17]. Community involvement in the developmental phase may, therefore, be particularly valuable, with high potential to identify indicators perceived as most relevant and to raise public awareness of local health issues that should be targeted for action [11].

Participatory approaches have already been useful in previous studies developing or prioritizing indicators in a small-scale context, e.g., in the development of comprehensive community health indicators in the USA [18] and Indigenous Health Indicators in the USA and Canada [19]. By employing participatory approaches, a greater number of viewpoints can support the study of complex issues [20] by generating knowledge in dialogue with involved stakeholder groups and integrating their various perspectives [21]. In particular, the research process can benefit from the view of ‘insiders’ [22] by involving those whose life or work is subject to the study [21] and who may be stakeholders, beneficiaries or users of the research [23]. Compared to merely researcher-led studies, a participatory approach can help to build stronger consensus and a shared vision with involved stakeholder groups [24], which increases the potential for acceptability and the translation of indicators into local decision-making and policy action [11].

We describe here the processes used in a pilot study to develop a neighborhood-level indicator system (referred to as a “neighborhood barometer”) for one pilot neighborhood in the German city of Mannheim. To meet users’ needs and thus increase the likelihood of sustainable transfer into practice, we partnered with representatives of the local city’s public health department and involved a broad range of stakeholders and possible end-users at multiple points throughout the development phase. Our objective was to design a web-based tool including neighborhood characteristics perceived as relevant and useful and, therefore, one that might support stakeholders at neighborhood (e.g., employees of the local community and headmasters of schools) and city levels (e.g., the local city’s public health department and the social welfare office) in further processes to identify and prioritize local needs and action points in the pilot neighborhood. To foster future work in this area, we also describe barriers and facilitators we encountered during the development and beta testing phases.

## 2. Materials and Methods

This pilot study was funded by the Ministry of Social Affairs, Health and Integration Baden-Wuerttemberg from mid-2018 to mid-2019. The research proposal was jointly drafted in collaborative partnership with representatives of the city’s public health department. Following the funding period, the city’s public health department and the public health research institute provided internal funds to continue the project.

### 2.1. Setting

Mannheim is a city in southwestern Germany that covers an area of 145 km^2^ with more than 320,000 residents [25,26]. For analyses, planning and administrative purposes, the city council subdivides a total of 38 different-sized (0.51–20.49 km^2^), geographically defined statistical units (referred to as “neighborhoods”) that represent coherent social spaces in the city [27]. Health-related interventions in these neighborhoods are defined and implemented by different stakeholders at the city- and neighborhood-level, namely city departments (e.g., the city’s public health department), individual local institutions (e.g., schools, kindergartens, youth centers and local self-help institutions) and interprofessional working groups (e.g., working groups for a family-friendly neighborhood). Several separate statistical reports by different city departments are available on selected topics, such as education, child health or social circumstances, to identify areas of social disadvantage and guide local decision-making. In neighborhoods identified as particularly disadvantaged, the city typically employs a neighborhood manager to run a neighborhood management office, a central contact point for residents and stakeholders. Typically, a neighborhood manager has a professional background in educational science, social science or in the areas of urban planning, local politics or public management and governance. Depending on funding structures, neighborhood managers are employed by a registered neighborhood management association or a non-profit organization. Their responsibilities include the coordination and implementation of activities and strategies to reduce disadvantages and to strengthen residents’ identification with the neighborhood. To date, five neighborhood managers have been assigned to neighborhoods throughout the city [28]. Through a mutual coordinator, the neighborhood managers are in regular contact with each other, as well as with stakeholders in their neighborhoods and on a city level.

To select a neighborhood that was suitable as a pilot region for developing a neighborhood-level indicator system, we held extensive discussions with partners at the city’s public health department centered on three main considerations: The pilot region was supposed to be a neighborhood that had not been the focus of health promotion projects in the past, where the local neighborhood manager expressed a high level of interest in proactively integrating health topics in their work, and where a specific interest existed in having a neighborhood assessment tool available. The neighborhood that was perceived to best meet these considerations covers an area of approximately 1.4 km^2^, with a population of almost 7500 inhabitants (i.e., a population density of about 5500 inhabitants per km^2^). At the time of study, the neighborhood population’s average age was 41.5 years, with a high proportion of inhabitants aged under 18 years (nearly 18.5%) compared to most other neighborhoods in Mannheim. In statistical reports, the neighborhood has been characterized as having above-average social problems (e.g., a high unemployment rate of above 8.5% and a high proportion of households at a high risk of poverty, such as above 4% of households with 3 or more children [26]).

### 2.2. Development Process

**Figure 1:**
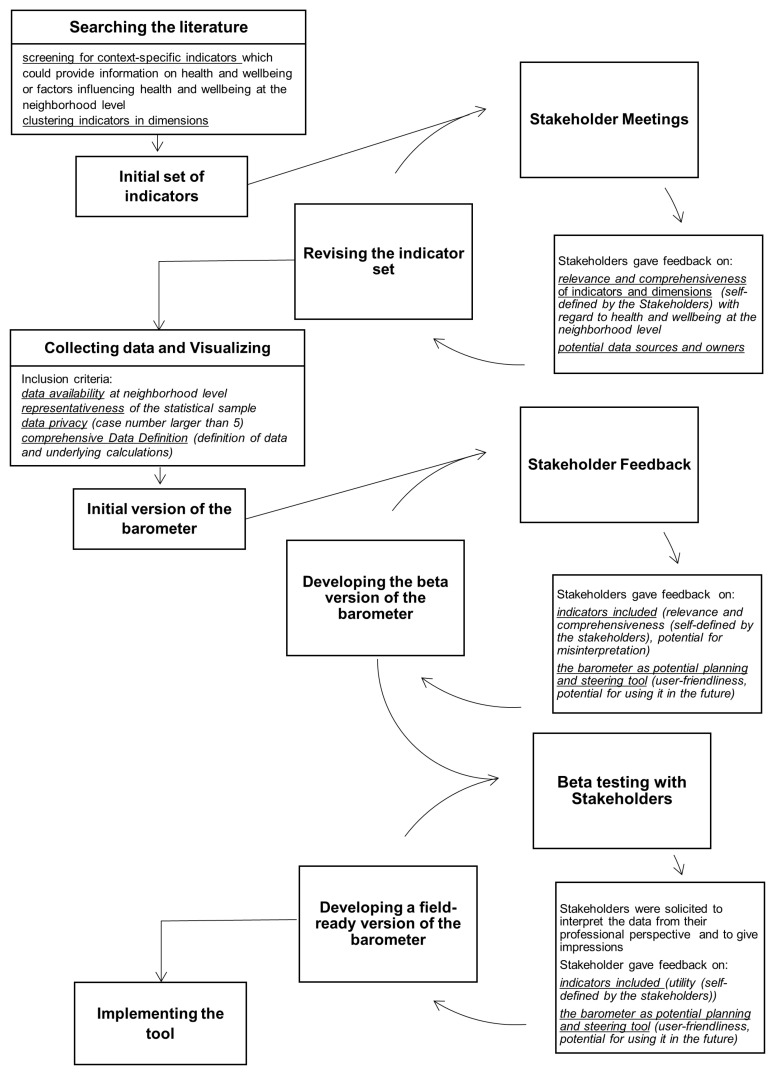
Steps in developing the neighborhood barometer.

In discussions with the city’s public health department, we chose to use an iterative approach consisting of several Plan-Do-Check-Act (PDCA) cycles [29] (Figure 1) that involved stakeholders at multiple points, described more fully below. We felt that this was an intuitive process with which community members would be familiar and which was respectful of time demands in contrast to other methods of consensus building (e.g., Delphi process [30]).

#### 2.2.1. Searching the Literature

Research team members (J.H.-K. and K.Ho.) conducted a non-systematic but extensive literature search to identify a broad range of health indicators used elsewhere in existing, mostly large-area systems to serve as a starting point for indicator development. The scientific databases PubMed/Medline and Web of Science were searched using keywords such as “health indicators”, “health reporting”, “health monitoring”, “wellbeing” and “quality of life” combined with the terms “city”, “community” and “neighborhood”. Considering that indicator systems are not always published in scientific journals, we also searched Google, Google Scholar and websites of various national and international organizations known for their expertise and practice in health monitoring, including the Robert Koch-Institute, State Institutes for Public Health, the WHO and the Organization for Economic Co-operation and Development. This search strategy was purpose-driven, with the goal of initiating discussions on thematic areas and individual indicators and providing a broad basis on which to develop the indicator system. Notably, in light of the timely scope of the project, the search was not intended to be as rigorous as for full systematic review.

The resulting literature was screened to identify potential neighborhood-level indicators that are directly or indirectly related to health and wellbeing. The potential indicators were collected and organized into clusters, which thematically group related indicators into various “dimensions” (e.g., “child health” or “education”). Initial thematically related clusters and suggestions for labels for each dimension were proposed by the research team (H.R., J.H.-K. and K.Ho.) and discussed in a group meeting with the city’s public health department representatives (K.He. and H.K.) until consensus was reached.

#### 2.2.2. Revising the Indicator Set

The initial set of indicators formed the basis for the first PDCA cycle (Figure 1). To explore whether indicators and dimensions previously identified in the literature search were likely to represent the health and wellbeing of residents in our pilot neighborhood, we invited a diverse group of city-level stakeholders who represented institutions already working directly with either the neighborhood manager in the pilot neighborhood or the city’s public health department. These included predominantly people with lower or middle management responsibilities (e.g., Institution Head or Department Head) influencing the situation in the neighborhood. They represented institutions including the social welfare office, the local statistics office, the local police department and institutions from the pilot neighborhood itself (e.g., school principals and the neighborhood manager) (Table 1). 

Elected representatives who may have certain competing interests were intentionally excluded from participation. We sought service providers from non-for-profit institutions who were best positioned to be aware of the needs and preferences of members of the pilot neighborhood. Inclusion criteria for participation in this process were having access to data on the neighborhood and/or active involvement in the pilot neighborhood, having connections within local networks and being a potential end-user of the neighborhood barometer. To broaden perspectives, stakeholders nominated others likely to work outside of the existing networks of the neighborhood manager or the city’s public health department for invitation to participate. Fourteen face-to-face meetings with 26 stakeholders (Table 1) lasting between one-half and two hours took place between June 2018 and February 2019. The number of participants varied from one to six stakeholders per session. Group meetings were scheduled by mail; individual meetings were offered for those who could not participate in a group meeting.

In these meetings, stakeholders were asked which dimensions and specific indicators they perceived most useful, using the indicator list we had created from the literature search. Furthermore, they were asked whether specific indicators and dimensions should be deleted or added to enhance comprehensiveness and relevance (both aspects self-defined), whether the initial assignment of clusters and labels was suitable, whether other potential data sources existed and who the data owners might be. The feedback obtained was discussed with our city’s public health department partners and resulted in agreement on an initial indicator set. Most participants knew each other from prior work, which created a collaborative work environment in which competing interests were not expected to impact the research process.

#### 2.2.3. Collecting of Data

In the next step, we contacted identified data owners (i.e., the local statistics office, the social welfare office, the city’s public health department, the youth welfare office, the police department and the local elementary school) to inquire whether desired data elements, aggregated or non-aggregated, were available for the pilot neighborhood. If those data were available, we requested the most recent data and data from 2010 to 2017 in order to facilitate trend recognition over time. We required that data for indicators were based on representative statistical samples and available for at least five residents to avoid identifiability. Even if it was not an inclusion criterion, all requested data were available free of charge. In order to assess validity, available data were checked for the validation of survey instruments used and the data owners’ provision of clearly defined data collection procedures and algorithms applied to the data. To provide guidance for future development of a neighborhood-level indicator system, data elements that did not meet these criteria or were not currently available but were considered relevant by one or more stakeholders were recorded as promising for incorporation into a future version of the neighborhood barometer. Reasons for excluding indicators were documented. We took various steps to establish the validity of the data. The data were checked for outlier values using SPSS Statistics 24 (IBM Corporation, Armonk, NY, USA). If unexpected values were identified, the data owner was consulted for clarification. Additional geospatial data in the form of street addresses and GPS coordinates for neighborhood-based resources or amenities were extracted from Google Maps and checked for validity by visiting each on site. We requested clear definitions for each indicator from the data owners or determined them ourselves after review of the data sources.

Although the process focused primarily on identifying actionable indicators directly related to health and wellbeing, we also wanted to ensure that relatively constant indicators indirectly related to health and wellbeing (e.g., percentage of households with children or gender structure) were included, as they might assist end-users’ in seeking highly specific information that could be cross-referenced with contextual characteristics of the neighborhood.

#### 2.2.4. Building the Visual Interface

We were interested in creating a tool that would be easy to update, that would be accessible to end-users without specialized software knowledge and that allowed visual representations of the data in complementary ways. It was planned that the city’s public health department would maintain the neighborhood barometer in the future. Thus, the research team decided jointly with representatives of the city’s public health department to select the software Tableau (Tableau Software LCC, Seattle, DC, USA) for several reasons: the ease of importing data from Excel files, straightforward tools for updating data, an intuitive user interface that did not require knowledge of programming languages and interactive functionality that allowed end-users to apply a variety of filters to create visual data reports on demand. For the data visualization in Tableau, we created one comprehensive Excel file filled with data on indicators and one Excel file including geospatial data. To facilitate data processing and to create linkages across Excel worksheets, we used a Tableau Add-In for Excel to pivot data reported by rows into columns. Based on the resulting Excel files, we created Tableau Worksheets and Dashboards that were finally merged to one Tableau Story. Unweighted data were displayed as absolute figures, percentages, proportions, average values or rates. Aggregated data for the entire city were provided per year as reference values.

#### 2.2.5. Developing the Beta Version

Refining the initial version of the barometer formed the basis for efforts in the second PDCA-cycle (Figure 1). The visual interface was shared with all previously participating stakeholders in February and March 2019. Feedback sessions took place in three face-to-face meetings with six individuals and by email with an additional three (Table 1). Each was asked to comment on the following areas: comprehensiveness, relevance and potential for misinterpretation of the chosen indicators, the overall user-friendliness of the visual interface and its potential as a planning and monitoring tool. Additionally, we asked those stakeholders who had provided data to comment on the accuracy of data presentation and the definitions provided. Feedback was recorded by team members (H.R., J.H.-K. and K.Ho.) using meeting notes, emails and protocols. Feedback responses were organized and summarized for each area investigated. Action steps resulting from this process and leading to development of a beta version (April–September 2019) included removing indicators with potential for misinterpretation and adding an introduction that specified aims and limitations of the barometer.

#### 2.2.6. Beta Testing

Beta testing and further refinement were at the core of the third PDCA-cycle (Figure 1). We held one virtual and three in-person feedback sessions between October 2019 and October 2020 with additional sessions limited by restrictions following the onset of the COVID-19 pandemic. Beta test participants included individuals or working groups, who were either engaged at the neighborhood level, had a neighborhood- or city-level leadership function, or had been involved in previous PDCA cycles (Table 1). In one case, we added discussion of the beta test as an agenda item to a previously scheduled meeting, whereas three other meetings were specifically devoted to the topic. In each meeting, we presented the visual interface for the beta version and solicited general impressions and interpretations of the data from participants’ diverse professional perspectives. Specifically, we asked for perceptions about the utility of the individual indicators presented and whether the neighborhood barometer as presented could be considered potentially useful. Feedback was recorded by team members (H.R., J.H.-K. and K.Ho.) using meeting notes which were summarized and discussed with representatives from the city’s public health department (K.He. and H.K.). As a result of these discussions, we added a description and geographical definition of the neighborhood. As no other feedback was provided, the research team and the city’s public health department agreed that the beta version was ready for implementation in the field.

#### 2.2.7. Implementing the Tool

Implementation plans started with a discussion with representatives from the city’s public health department about which stakeholder groups might represent potential end-users. The next steps were interrupted by measures to contain the COVID-19 pandemic in Germany and the involvement of representatives from the city’s public health department in local pandemic management. They included an introductory session to the beta version for these potential end-users. Going forward, plans include use of future PDCA cycles to address verbal and written comments and feedback on specific aspects, general utility of the neighborhood barometer and confirmation of the extent to which indicators reflect the intended characteristics. Actual use of the web-based interface will be determined by monthly website usage statistics.

#### 2.2.8. Updating the Tool

As measures to contain the COVID-19 pandemic in Germany became weaker and the involvement of our partners in local pandemic management decreased at the end of the writing of this manuscript and during the revision phase, representatives of the city’s public health department were able to update the data to the years 2018, 2019, 2020 and 2021. They contacted data owners again and updated the existing Excel file with available data. Regular updates from the city’s public health department are planned.

#### 2.2.9. Uncovering Facilitators and Barriers to Creating a Neighborhood Barometer

To identify lessons learned, potential barriers and facilitators in creating a neighborhood barometer, we regularly reviewed and extensively discussed the process, impressions and experiences documented in protocols of project meetings within the research team (H.R., J.H.-K. and K.Ho.). We defined facilitators as factors that simplified the process and barriers as factors that hindered either the development process or the implementation phase.

## 3. Results

### 3.1. The Field-Ready Version of the Neighborhood Barometer

At this stage, the iterative, participatory approach resulted in agreement on a field-ready version consisting of 86 indicators grouped into eight dimensions, which we labelled population structure, population development, household structure, material wellbeing (economics), education, family and upbringing, child health and personal security. The data displayed in the barometer monitor relevant indicators over time and in comparison to the entire city of Mannheim. Through this process, we identified and subsequently mapped geospatial data for 43 amenities thought to be related to health or wellbeing in our pilot neighborhood.

The selected indicators were largely based on aggregated registration data from local offices or agencies (i.e., the local statistics office, the youth welfare office and the police department). Other non-aggregated data were obtained from the early childhood intervention service “Welcome to Life” by the youth welfare office and from school entry screenings conducted by the city’s public health department, in which previously validated instruments were applied (e.g., body weight measurement using standardized scales). Data derived from nonvalidated instruments were excluded from the field-ready version of the neighborhood barometer. Detailed information on indicators included in the field-ready version of the neighborhood with information on the data owner, received data and data preparation for visualization can be found in Supplementary Table S1. Due to the inclusion of predominantly aggregated data, cross-tabulation between indicators is not possible. In addition, not all data were available for an update due to the COVID-19 pandemic (e.g., no school entry screenings took place during the pandemic).

**Figure 2:**
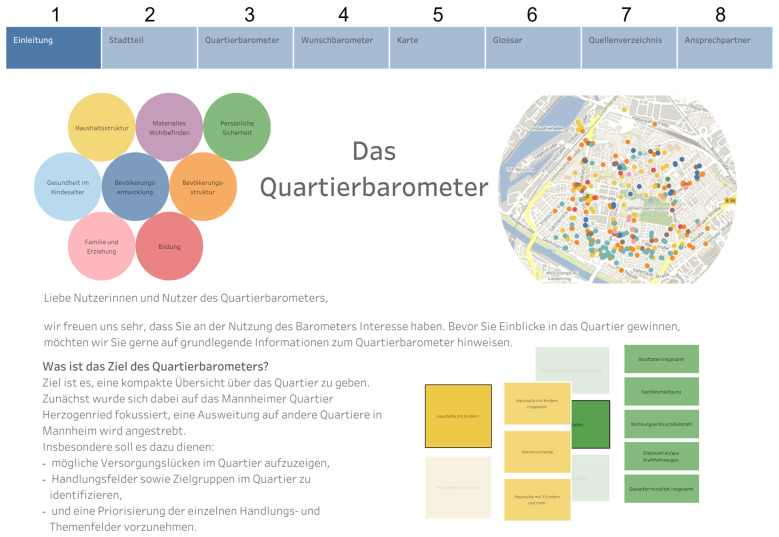
Illustration of the visual interface (in German).

As a result of the feedback loops with stakeholders and ongoing discussions and decisions made with representatives from the city’s public health department, the website for the neighborhood barometer was organized using tabs serving different purposes (Figure 2), namely introduction (1), neighborhood (2), neighborhood barometer (3), parking lot (4), map (5), glossary (6), references (7) and contact persons (8).

**Figure 3:**
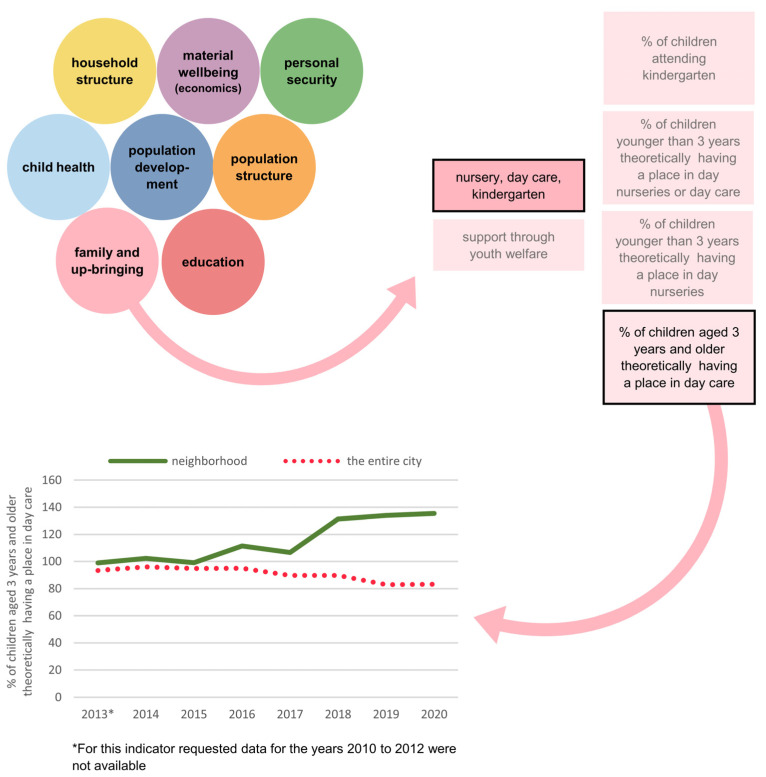
Example application of the neighborhood barometer.

An introduction (1) [Einleitung] clarified the intended purpose, the aims, the opportunities, the limits and the structure of the neighborhood barometer in response to stakeholder feedback. A description and geographical definition of the neighborhood (2) [Stadtteil] specified the underlying population characteristics to maximize transparency, as some end-users might be unaware of the borders of the statistical unit comprising the pilot neighborhood. An overview of the data contained (3) [Quartierbarometer] is illustrated by a series of bubbles identifying each dimension (i.e., population structure, population development, household structure, material wellbeing, education, family and upbringing, child health and personal security) to facilitate exploration of the data (Figure 2). An example of the application of the neighborhood barometer for a potential user interested in family and upbringing indicators is presented in Figure 3.

As stakeholder meetings resulted in the identification of an additional 123 indicators perceived as relevant but lacking readily available data, a “parking lot” of ideas was provided (4) [Wunschbarometer] to enable retention of this information and to guide the possible direction of future data collection and analytical opportunities in the piloted neighborhood (Supplementary Table S2). For example, various environmental indicators (perceived heat stress, perceived air quality, perceived noise pollution and total share of green areas) were identified as important and relevant for the neighborhood context but lacking data for inclusion in the barometer. They were, therefore, added to the parking lot. 

As feedback from several participants stressed the importance of being able to visualize the physical infrastructure of the neighborhood and the need to identify potential shortfalls in particular amenities, we provided a visual display of selected features in map format (5) [Karte], which could be filtered on demand. Additionally, a glossary (6) [Glossar] containing definitions and data resources, a list of references (7) [Quellenverzeichnis] and contact persons for questions and suggestions (8) [Ansprechpartner] were added to the website to maximize transparency (Figure 2).

### 3.2. Facilitators and Barriers

Stakeholder involvement and engagement was verified as a key facilitator in creating a neighborhood barometer through review of meeting minutes and project diaries. This aspect of the development process added value, as it uncovered relevancies that were not obvious to the research team purely on the basis of previously published literature identified through the literature search. For example, nearly 40 indicators not identified in the literature were included due to stakeholders’ perceptions (e.g., “proportion of children who can/cannot swim in primary school” as stakeholders had concerns about this topic). Moreover, 25 indicators from the literature search were excluded as stakeholders raised concerns about misinterpretations or the lack of informative value to an extent the research team had not anticipated (e.g., “number of private cars per household” was felt to be neither an accurate indicator for material wellbeing nor for mobility in this neighborhood). 

Investing time and holding meetings in person appeared to further facilitate the developmental process. Upon reflection, the perceived quality of interaction seemed greater in meetings dedicated entirely to discussion of the neighborhood barometer compared with those in which it was discussed as one of several agenda items. Additionally, meetings in person compared with those held online resulted in more extensive discussions and active interpretation of the data presented in the barometer and a greater quantity of feedback, as reflected by the number of comments, reactions and amount of time devoted to the discussions. Review of study notes also suggested that the continuous and substantial nature of collaboration with our partners from the city’s public health department facilitated the process. For example, they regularly participated in project meetings, identified and enabled access to members of an extensive existing network of stakeholders and dedicated personnel resources to maintenance and updating the neighborhood barometer in the future. 

The primary barrier we encountered was the onset of the COVID-19 pandemic and related containment measures, which halted plans for implementing the beta version of the neighborhood barometer in the proposed pilot neighborhood. For example, face-to-face group meetings were prohibited in Germany at that time. Priorities among stakeholders also changed dramatically during this time, leaving participants with relatively few opportunities to familiarize themselves with a new indicator system in the face of more pressing tasks. In addition, our partners at the city’s public health department were highly involved in local pandemic management, which was prioritized over the implementation and updates of the barometer for a long time.

Another important barrier complicating the development of a pilot neighborhood barometer was the unavailability of data endorsed by stakeholders as potentially informative. For example, data for more than 100 indicators in different areas were not routinely collected by any city agency or non-governmental organization. Alternatively, data on many indicators of interest were available, but not at the neighborhood level or only for other kinds of administrative units such as constituencies or school catchment areas. Finally, some useful data elements were available at a neighborhood level but with case numbers too small to guarantee data privacy. 

## 4. Discussion

In close collaboration with partners at our local public health department and by soliciting and incorporating stakeholder ideas and feedback, we designed and developed a field-ready neighborhood-level indicator system of health and wellbeing tailored to the local and end-users’ needs in a neighborhood in Mannheim, Germany. Following several PDCA cycles with multiple feedback loops each, we created a user-friendly, web-based tool that presents data in various, complementary ways in order to meet the differing informational needs of a diverse group of stakeholders in this neighborhood. Stakeholder feedback supported the inclusion of indicators deemed relevant, a suitable grouping scheme of indicators in various dimensions, and comprehensibility of definitions applied. In addition, it allowed us to critically appraise indicators and investigate their potential for misinterpretation. 

Using an iterative participatory approach, we were able to incorporate stakeholders’ preferences, professional knowledge and feedback and gained a clearer sense of the context for data presented in the barometer at multiple points during the developmental process. In line with benefits reported in previous research [11], we experienced that extensive meetings with a heterogeneous group of stakeholders from various disciplines fostered intersectoral exchange throughout the developmental phase. The collaboration with representatives of the city’s public health department who are local key actors in monitoring residents’ health enabled us to better understand local needs and stakeholders’ feedback. In addition, we benefited from the city’s public health department as a door opener to stakeholders at the neighborhood and city levels. This collaborative relationship, which began with jointly writing the funding application, resulted in a high level of transparency during the development process, mutual decision-making, and a field-ready version of the barometer with broad support from involved stakeholders. Additionally, the collaboration served as a foundation for joint efforts on other currently ongoing projects, demonstrating the sustainability of this approach. 

Overall, the process we followed provides strong evidence for the potential value and synergy that may arise from collaborations between public health science and local health departments. To leverage the full potential of these collaborations, discussion on ways to support and build connections between disciplines will be important. In the German state of Baden-Wuerttemberg, current efforts have taken shape in the form of the Center for Public Health and Health Services Research, established in 2019 [31], to support linkages between university medicine, health-care research and health services. Opportunities for similar formalizations of local research collaborations are anticipated in other settings in which this approach might be used, with high potential to respond to local public health needs.

Previous research suggests that successful collaboration benefits significantly from time devoted to relationship building despite the increased burdens this may pose on those involved [32,33]. Finding a common language, developing shared goals, and acknowledging mutual and individual concerns, however, represent elements of a strategy that respects differing work cultures and that may counterbalance the short-term costs of relationship building. From our experience in this study, we suggest that time for these aspects of relationship building should be scheduled as formal activities to be supported by funding agencies. 

We were able to facilitate a process in which stakeholders agreed on a set of indicators perceived as relevant for identifying and prioritizing local needs and action points in the target neighborhood. The neighborhood barometer was largely based on registration data from local offices or agencies that guaranteed the accuracy of data at the neighborhood level. However, due to the lack of data availability for some indicators, it was not possible to include many health-related data elements of interest, which were collated in a repository (the “Parking Lot”, Supplementary Table S2). The Parking Lot shows that in particular, stakeholders missed information on health in all age groups (i.e., children, adolescents, adults and seniors), subjective population wellbeing (e.g., self-rated health status and life satisfaction), living conditions (e.g., perceived heat stress and perceived air quality) and participation and involvement (e.g., neighborhood cohesion and electoral turnout in different elections). Considering the multidimensionality of health [34,35] and the associations between diverse characteristics of neighborhood environments and different dimensions of health [36], our study suggests that at least at the local level, the current availability of data appears insufficient in reflecting the interconnectedness of place and health. 

We hope that the awareness of this complexity and desire for more specific and granular data among stakeholders, as indicated by their feedback obtained in the development process, will lead to more comprehensive efforts in routinely recording and collecting neighborhood-level data for analysis beyond the current standard. Increasing digitalization holds potential to open access to usable, new data sources such as social media platforms and geolocation data available through mobile devices [8]. In addition, primary data collection using neighborhood surveys can generate a deeper understanding of residents’ perceptions of their needs and thereby support the definition and identification of local priorities [37]. Moreover, primary data collection would enable the previously missing function of cross-tabulation between indicators and dimensions that various stakeholders desired. Accordingly, a further concentration of resources and skill-building for the optimal use of existing data sources and opportunities for the development of new data sources are needed. At present, there are no established routines in place at the municipal level to collect data identified by the participants of this pilot study. Discussions are currently underway and will be more thoroughly explored following completion of a field test. The Parking Lot provides additional insight into local stakeholder preferences and might provide guidance for developers and funders of a neighborhood-level indicator system for monitoring and surveillance.

Even though the processes described here appear useful for researchers, community planners and policy makers who are planning to fund, develop or revise neighborhood-level indicator systems elsewhere, a few limitations should be noted. Although the indicators selected by stakeholders in the pilot neighborhood in Mannheim are shaped by local needs, preferences and data availability, the contribution of this work is best reflected by the processes used in identifying health-related indicators endorsed by potential end-users and through the recognition of facilitators and barriers in the processes used to identify them. A non-systematic literature search to identify an initial set of indicators (1st PDSA) was chosen for pragmatic reasons (the limited funding period); this may not have identified all indicators covered in international literature, which might have lowered the odds of their consideration during the participatory development process. In addition, the majority of stakeholders in this study work with children and young people, which may have influenced the selection of indicators toward a focus on child health. The limited availability of data for integration in the barometer enhanced this focus on child health, which somewhat shifts when all potential indicators from the Parking Lot (Supplementary Table S2) are considered. For example, environmental indicators of health and wellbeing were regarded as important but could only be added to the Parking Lot. Furthermore, our study benefitted from pre-existing networks of the city’s public health department and the neighborhood management. In the absence of such networks, researchers and indicator developers should consider the potential for difficulties that may arise in establishing new contacts and in the consensus process, which may require additional time and efforts.

Given our inability to conduct a field trial during the pandemic, we were not able to fully establish the validity of the indicators that have been endorsed as being relevant by the neighborhood stakeholders. Accordingly, although outside the funded timeline, the barometer will be field-tested and future PDCA cycles will be conducted to further refine the instrument and tailor it to potentially unanticipated local needs and logistical challenges. At the time of writing, our partners in the city’s public health department were in the process of updating the data of the included indicators. In addition, the development of the barometer and especially the Parking Lot resulted in a survey in the pilot neighborhood to collect information that was unavailable when the barometer was developed. The upcoming analysis and integration of survey results into the neighborhood barometer will be used to further enhance the instrument and will be subject to another publication. Based on these survey results, we anticipate further discussions and the implementation of the neighborhood barometer, which is still the goal of all involved. Upon implementation and evaluation of the tool, ambitions include the extension of the instrument to other neighborhoods in Mannheim and its integration in local decision-making processes. Future work should consider the development of small-area indicator sets using participatory stakeholder approaches in other geographic areas, where adaptations of our barometer and Parking Lot may serve as a starting point to discuss local need.

## 5. Conclusions

We present an iterative participatory approach, characterized by stakeholder involvement and a strong collaboration with the local public health department that resulted in the development of and agreement on a field-ready small-area indicator system of health and wellbeing for a pilot neighborhood in Germany, tailored to local and users’ needs. Namely, the agreed system includes 86 indicators across eight domains (population structure, population development, household structure, material wellbeing, education, family and upbringing, child health and personal security), with a further 123 indicators excluded from the instrument due to data structures. The process described here contributes to and should encourage further work in developing meaningful, useful and sustainable neighborhood-level tools that can be used to monitor and promote health and wellbeing. Our work also identified the lack of non-aggregated health data useful for neighborhood monitoring and surveillance in the pilot neighborhood in Mannheim. Collecting these data would enable the study of population health at a neighborhood level (e.g., through cross-tabulation of indicators) and may contribute to identifying local health needs for targeted allocation decisions and policy development. By collaboratively developing and implementing similar tools in other regions, local stakeholders can actively support the identification and reduction of sub-national health inequalities.

## Figures and Tables

**Table 1:** Numbers of stakeholders involved by affiliation for each feedback loop.

	*n* in Stakeholder Meetings	*n* in Stakeholder Feedback	*n* in Beta Testing
city-level:			
youth welfare office	5	3	
citizen involvement	1	1	
social welfare office	2		4
city’s public health department	4		2
education office	1	1	
local statistics office	2	1	
neighborhood-level:			
local elementary school (i.e., school management, social worker)	3	1	1
local integrated comprehensive school	1		1
kindergartens			2
health and patient advisory service [Gesundheitstreffpunkt]	1	1	
neighborhood management	1		1
housing association	1		1
youth center	1		1
local police department	2	1	
neighborhood library	1		

## Data Availability

Data sharing not applicable. No new data were created or analyzed in this study. Data sharing is not applicable to this article.

## Supplementary Materials

The following supporting information can be downloaded at: https://www.mdpi.com/article/10.3390/ijerph20021456/s1, Table S1 List of indicators perceived as relevant by stakeholders of a single targeted neighborhood and thus chosen to be included in a field-ready version of the neighborhood barometer. Table S2 “The parking Lot”: Indicators desired by participants for which no data were available and could therefore not be incorporated in the field-ready version of the neighborhood barometer.
