# Supplementary material for: An Iterative, Participatory Approach to Developing a Neighborhood-Level Indicator System of Health and Wellbeing
Source: Int J Environ Res Public Health. 2023 Jan 13;20(2):1456. doi: 10.3390/ijerph20021456 (PMC9859574; doi:10.3390/ijerph20021456)
Supplement: Supplementary file 1 [file ijerph-20-01456-s001.zip › ijerph-2152712-supplementary.pdf]

Table S1 List of indicators perceived as relevant by stakeholders of a single targeted neighborhood and thus chosen to be included in a field-ready version of the neighborhood barometer.

**dimensions**	**categories**	**indicators**	**data owner**	**received data**	**data preparation for visualization**
population structure	total population	absolute figures for the resident population (main and secondary residence)	the local statistics office	aggregated register data: absolute figures for the resident population (main and secondary residence)	no calculation of new variables required
		absolute figures for the main resident population	the local statistics office	aggregated register data: absolute figures for the main resident population	no calculation of new variables required
		absolute figures for the secondary resident population	the local statistics office	aggregated register data: absolute figures for the main resident population<br><br>absolute figures for the resident population (main and secondary residence)	calculation of the absolute figures for the secondary resident population *(resident population – main resident population)*
	gender structure	% and absolute figures for gender distribution	the local statistics office	aggregated register data:	calculation of the percentage

			absolute figures for female and male main resident population	_(female main resident population/ main resident population) * 100_<br>_(male main resident population/ main resident population) * 100_
age structure	% of the population < 18 years	the local statistics office	aggregated register data: absolute figures for the main resident population < 18 years<br>absolute figures for the main resident population	calculation of the percentage _(main resident population <18 years/ main resident population) * 100_
	% of the population ≥ 65 years	the local statistics office	aggregated register data: absolute figures for the main resident population ≥ 65 years<br>absolute figures for the main resident population	calculation of the percentage _(main resident population ≥_ 65 years / _main resident population) * 100_

youth dependency ratio[1] (% of working-age population)	the local statistics office	aggregated register data: absolute figures for the main resident population 0 - 19 years<br><br>absolute figures for the main resident population 20 - 64 years	calculation of the youth dependency ratio *(main resident population* 0 - 19 years / *main resident population 20 – 64 years) * 100*
old-age dependency ratio[2] (% of working-age population)	the local statistics office	aggregated register data: absolute figures for the main resident population ≥ 65 years<br><br>absolute figures for the main resident population 20 - 64 years	calculation of the old-age dependency ratio *(main resident population* ≥ 65 years / *main resident population 20 – 64 years) * 100*
dependency ratio[3] (% of working-age population)	the local statistics office	aggregated register data:	calculation of the old-age dependency ratio

[1] ratio of younger dependents - people aged 0 to 20 years – to the working-age population aged between 20 and 64 years
[2] ratio of older dependents - people aged 65 years and older – to the working-age population aged between 20 and 64 years
[3] ratio of dependents - people aged 0 to 20 years or 65 years and older – to the working-age population aged between 20 and 64 years

			absolute figures for the main resident population 0 - 19 years<br><br>absolute figures for the main resident population 20 - 64 years<br><br>absolute figures for the main resident population ≥ 65 years	*((main resident population 0 - 19 + main resident population ≥ 65 years) / main resident population 20 – 64 years) * 100*
migration background	% and absolute figures for the population with a migration background[4]	the local statistics office	aggregated register data: absolute figures for the main resident population with a migration background	calculation of the percentage *(main resident population with a migration background / main resident population) * 100*

[4] persons without German citizenship, naturalized Germans and ethnic German repatriates as well as children with at least one parent with a migration background

	% of the population with a migration background[4] <18 years of age	the local statistics office	aggregated register data: absolute figures for the main resident population with a migration background < 18 years<br><br>absolute figures for the main resident population < 18 years	calculation of the percentage *(main resident population with a migration background < 18 years / main resident population <18 years) * 100*
	% of the population with a migration background without German citizenship	the local statistics office	aggregated register data: absolute figures for the main resident population without German citizenship<br><br>absolute figures for the main resident population with a migration background	calculation of the percentage *(main resident population without German citizenship / main resident population with a migration background) * 100*
	% of population without German citizenship <18 years of age	the local statistics office	aggregated register data: percentage of population without German citizenship <18 years of age	no calculation of new variables required

population development	natural population development	fertility rate[5]	the local statistics office	aggregated register data:<br>absolute figures for births<br>absolute figures for female main resident population 15 – 44 years	calculation of the fertility rate<br>_(births / female main resident population 15 – 44 years) * 1000_
		natural population balance[6]	the local statistics office	aggregated register data:<br>absolute figures for births<br>absolute figures deaths<br>absolute figures for the main resident population	calculation of the natural population balance<br>_((births – deaths) / main resident population) * 1000_
	migration	migration balance[7]	the local statistics office	aggregated register data:<br>absolute figures for immigrants<br>absolute figures for emigrants	calculation of the migration balance<br>_((immigrants – emigrants) * 1000) / main resident population_

[5] the number of live births per 1.000 women aged 15 to 44 years
[6] the difference between the number of deaths and live births per 1.000 people
[7] the difference between immigrants and emigrants per 1.000 people

				absolute figures for the main resident population	
		% of the population born in Mannheim	the local statistics office	aggregated register data: absolute figures for the main resident population born in Mannheim<br><br>absolute figures for the main resident population	calculation of the percentage *(main resident population born in Mannheim / main resident population) * 100*
		average duration of residence at the same address	the local statistics office	aggregated register data: average duration of residence at the same address	no calculation of new variables required
household structure	total households	absolute figures for private households	the local statistics office	aggregated register data: absolute figures for private households of the resident population (main and secondary residence)	no calculation of new variables required
	households with children	% of total households with children	the local statistics office	aggregated register data: absolute figures for private households of the resident	calculation of the percentage

		population (main and secondary residence) with children<br><br>absolute figures for private households of the resident population (main and secondary residence)	*(private households of resident population with children / private households of resident population) * 100*
% of single parents (% of households with children)	the local statistics office	aggregated register data: absolute figures for private households of the resident population (main and secondary residence) with children and single parents<br><br>absolute figures for private households of the resident population (main and secondary residence) with children	calculation of the percentage *(private households of resident population with children and single parents / private households of resident population with children) * 100*

	% of households with ≥ 3 children (% of households with children)	the local statistics office	aggregated register data: absolute figures for private households of the resident population (main and secondary residence) with ≥ 3 children<br><br>absolute figures for private households of the resident population (main and secondary residence) with children	calculation of the percentage *(private households of resident population with* ≥ 3 *children/ private households of resident population with children) * 100*
households without children	% of single-person households	the local statistics office	aggregated register data: absolute figures for single-person private households of the resident population (main and secondary residence)<br><br>absolute figures for private households of the resident	calculation of the percentage *(single-person private households of resident population/ private households of resident population) * 100*

				population (main and secondary residence)	
material wellbeing (economics)	employment / unemployment	total employment rate[8] (% of working-age population)	the local statistics office	data aggregated by the local statistics office (original data basis: Bundesagentur für Arbeit (the German Federal Employment Agency)): absolute figures for persons employed subject to social security contributions<br><br>aggregated register data: absolute figures for the main resident population 15 - 65 years	calculation of the total employment rate *(persons employed subject to social security contributions / main resident population 15 - 65 years) * 100*
		employment rate in the population without German citizenship	the local statistics office	data aggregated by the local statistics office (original data basis: Bundesagentur für Arbeit	calculation of the employment rate in the population

[8] ratio of persons employed subject to social security contributions (independent of qualification possessed) to the working age population – people aged between 15 and 65 years

		(the German Federal Employment Agency)): absolute figures for persons employed subject to social security contributions without German citizenship<br><br>aggregated register data: absolute figures for the main resident population 15 - 65 years without German citizenship	without German citizenship *(persons employed subject to social security contributions without German citizenship/ main resident population 15 - 65 years without German citizenship) * 100*
female employment rate	the local statistics office	data aggregated by the local statistics office (original data basis: Bundesagentur für Arbeit (the German Federal Employment Agency)): absolute figures for women employed subject to social security contributions	calculation of the female employment rate *(women employed subject to social security contributions without German citizenship/ female main resident*

		aggregated register data: absolute figures for the female main resident population 15 - 65 years	*population 15 - 65 years) * 100*
total unemployment rate[9] (% of working-age population)	the local statistics office	data aggregated by the local statistics office (original data basis: Bundesagentur für Arbeit (the German Federal Employment Agency)): absolute figures for the unemployed<br><br>aggregated register data: absolute figures for the main resident population 15 - 65 years (excluded refugees)	calculation of the total unemployment rate *(unemployed/ main resident population 15 - 65 years excluded refugees) * 100*
unemployment rate in the age group 15 - 24 years	the local statistics office	data aggregated by the local statistics office	calculation of the unemployment rate

[9] ratio of unemployed to the working age population – people aged between 15 and 65 years

		(original data basis: Bundesagentur für Arbeit (the German Federal Employment Agency)): absolute figures for unemployed 15 – 24 years<br><br>aggregated register data: absolute figures for the main resident population 15 - 24 years (excluded refugees)	in the age group 15 - 24 years *(unemployed 15 – 24 years/ main resident population 15 - 24 years excluded refugees) * 100*
unemployment rate in the age group 25 - 65 years	the local statistics office	data aggregated by the local statistics office (original data basis: Bundesagentur für Arbeit (the German Federal Employment Agency)): absolute figures for unemployed 25 – 65 years<br><br>aggregated register data:	calculation of the unemployment rate in the age group 25 - 65 years *(unemployed 25 – 64 years/ main resident population 25 - 64 years excluded refugees) * 100*

			absolute figures for the main resident population 25 - 65 years (excluded refugees)	
recipients of social benefits	% of individuals receiving Minimum Income Benefits	the social welfare office	data aggregated by the social welfare office (original data basis: Bundesagentur für Arbeit (the German Federal Employment Agency)): % of individuals receiving Minimum Income Benefits	no calculation of new variables required
	% of individuals < 65 years receiving unemployment benefits	the social welfare office	data aggregated by the social welfare office (original data basis: Bundesagentur für Arbeit (the German Federal Employment Agency)): % of individuals < 65 years receiving unemployment benefits	no calculation of new variables required

% of unemployable individuals living in a household in need of social benefits[10] according to book II of the German Social Code	the social welfare office	data aggregated by the social welfare office (original data basis: Bundesagentur für Arbeit (the German Federal Employment Agency)): unemployable individuals living in a household in need of social benefits according to book II of the German Social Code	no calculation of new variables required
% of single-parent households in need of social benefits[10] according to book II of the German Social Code	the social welfare office	data aggregated by the social welfare office (original data basis: Bundesagentur für Arbeit (the German Federal Employment Agency)): % of single-parent households in need of social benefits according to	no calculation of new variables required

[10] at least one employable household member is eligible for social benefits

				book II of the German Social Code	
		% of individuals > 65 years receiving basic subsistence income for the elderly	the social welfare office	data aggregated by the social welfare office (original data basis: Bundesagentur für Arbeit (the German Federal Employment Agency)): % of individuals > 65 years receiving basic subsistence income for the elderly	no calculation of new variables required
education	elementary school – % of students with a migration background	% of students with migration background[4] in elementary school	the local elementary school	aggregated school statistics: absolute figures for students with migration background<br><br>absolute figures for students	calculation of the percentage *(students with migration background/ students) * 100*
		% of students without German citizenship in elementary school	the local elementary school	aggregated school statistics: absolute figures for students without German citizenship	calculation of the percentage

		absolute figures for students	*(students without German citizenship/ students) * 100*
% of students with migration background but with German citizenship in elementary school	the local elementary school	aggregated school statistics: absolute figures for students with migration background<br><br>absolute figures for students without German citizenship<br><br>absolute figures for students	calculation of the absolute figures for students with migration background but with German citizenship *students with migration background - students without German citizenship*<br><br>calculation of the percentage *(students with migration background but with*

				*German citizenship/ students) * 100*
elementary school – recommendations and transition to secondary school[11]	% of elementary school students with a transfer recommendation for lower secondary education ("Hauptschule")[12]**Error! Bookmark not defined.**	the local elementary school	aggregated school statistics: absolute figures for students with a transfer recommendation for lower secondary education ("Hauptschule")<br><br>absolute figures for students in grade 4	calculation of the percentage *(students with a transfer recommendation for lower secondary education ("Hauptschule") / students in grade 4) * 100*
	% of elementary school students with a transfer recommendation for intermediary secondary school ("Realschule")[13]**Error! Bookmark not defined.**	the local elementary school	aggregated school statistics: absolute figures for students with a transfer recommendation for intermediary secondary school ("Realschule") **Error! Bookmark not defined.**	calculation of the percentage *(students with a transfer recommendation for intermediary secondary school*

[11] In Germany the transfer recommendation the school gave and the school choice can differ
[12] lower secondary school level, usually comprising the grades 5 to 9
[13] lower secondary school level, usually comprising the grades 5 to 10

		absolute figures for students in grade 4	*(“Realschule”)/ students in grade 4) * 100*
% of elementary school students with a transfer recommendation for high school (“Gymnasium”)[14] **Error! Bookmark not defined.**	the local elementary school	aggregated school statistics: absolute figures for students with a transfer recommendation for high school (“Gymnasium”) **Error! Bookmark not defined.** absolute figures for students in grade 4	calculation of the percentage *(students with a transfer recommendation for high school (“Gymnasium”)/ students in grade 4) * 100*
% of elementary school students without transfer recommendation (e.g., pupils with special needs like pupils without basic knowledge of German language)	the local elementary school	aggregated school statistics: absolute figures for students without transfer recommendation **Error! Bookmark not defined.**	calculation of the percentage *(students without transfer recommendation/ students in grade 4) * 100*

[14] upper secondary school level, usually comprising the grades 5 to 12 or 5 to 13, academic secondary education is required to enter stages of tertiary education

		absolute figures for students in grade 4	
% of transitions from elementary school to lower secondary school ("Hauptschule")[11]**Error! Bookmark not defined.**	the local elementary school	aggregated school statistics: absolute figures for transitions from elementary school to lower secondary school ("Hauptschule")**Error! Bookmark not defined.** absolute figures for students in grade 4	calculation of the percentage *(transitions from elementary school to lower secondary school ("Hauptschule")/ students in grade 4) * 100*
% of transitions from elementary school to intermediary secondary school ("Realschule")[12]	the local elementary school	aggregated school statistics: absolute figures for transitions from elementary school to intermediary secondary school ("Realschule")**Error! Bookmark not defined.** absolute figures for students in grade 4	calculation of the percentage *(transitions from elementary school to intermediary secondary school ("Realschule")/ students in grade 4) * 100*

% of transitions from elementary school to high school ("Gymnasium")[13]	the local elementary school	aggregated school statistics: absolute figures for transitions from elementary school to high school ("Gymnasium") **Error! Bookmark not defined.**<br><br>absolute figures for students in grade 4	calculation of the percentage *(transitions from elementary school to high school ("Gymnasium")/ students in grade 4) * 100*
% of transitions from elementary school to community school ("Gemeinschaftsschule")[15]	the local elementary school	aggregated school statistics: absolute figures for transitions from elementary school to community school ("Gemeinschaftsschule") **Error! Bookmark not defined.**<br><br>absolute figures for students in grade 4	calculation of the percentage *(transitions from elementary school to community school ("Gemeinschafts-schule")/ students in grade 4) * 100*

[15] lower and secondary school level, usually comprising three different courses of education ("Hauptschule", "Realschule" and "Gymnasium")

	% of transitions from elementary school to another type of school (e.g. integrated comprehensive school)[16]	the local elementary school	aggregated school statistics: absolute figures for transitions from elementary school to another type of school **Error! Bookmark not defined.**<br><br>absolute figures for students in grade 4	calculation of the percentage *(transitions from elementary school to another type of school/ students in grade 4) * 100*
elementary school - language support needs	% of children with language support needs, surveyed as part of the school entry examination	the city public health department	non-aggregated data sets of the school entry examination:<br>results of the Heidelberger Auditives Screening in der Einschulungsuntersuchung (HASE), (a validated German screening procedure) for every child in the data sets	calculation of a new variable "*child with language support needs*" using threshold values of HASE<br><br>calculation of the percentage *(children with language support needs/ children*

[16] lower and secondary school level, comprising three different courses of education ("Hauptschule", "Realschule" and "Gymnasium")

					*examined in the school entry examination) * 100*
		% of children with language support needs determined by the framework of the Diagnostic Spelling Test in the elementary school years (grades 1 to 4)	the local elementary school	aggregated school statistics: percentage of students with language support needs determined by the framework of the Diagnostic Spelling Test	no calculation of new variables required
family and upbringing	nursery, day care, kindergarten	% of children attending kindergarten	the city public health department	non-aggregated data sets of the school entry examination: information on the name of the attended kindergarten for every child in the data sets	calculation of a new variable "*child attended kindergarten*"<br><br>calculation of the percentage *(children attended kindergarten/ children examined in the school entry examination) * 100*

	% of children aged 3 years and older theoretically[17] having a place in day care	the youth welfare office	aggregated youth welfare office statistics: % of children aged 3 years and older theoretically having a place in day care	no calculation of new variables required
	% of children aged younger than 3 years theoretically[17] having a place in day nurseries or day care	the youth welfare office	aggregated youth welfare office statistics: % of children aged younger than 3 years theoretically having a place in day nurseries or day care	no calculation of new variables required
	% of children aged younger than 3 years theoretically[17] having a place in day nurseries	the youth welfare office	aggregated youth welfare office statistics: % of children aged younger than 3 years theoretically having a place in day nurseries	no calculation of new variables required
support through youth welfare	total number of educational support[18] per 1.000 inhabitants aged 0 to 21 years	the youth welfare office	aggregated youth welfare office statistics:	no calculation of new variables required

[17] the availability of a place in the neighborhood independent of the actual utilization
[18] child and youth welfare services including services like educational guidance or full-time foster care

				total number of educational support per 1.000 inhabitants aged 0 to 21 years	
child health	health-related behavior	% of elementary school-aged children able to swim	the local elementary school	aggregated school statistics: percentage of students able to swim in grade 3	no calculation of new variables required
		% of elementary school-aged children able to ride a bicycle safely	the local elementary school	aggregated school statistics: percentage of students able to ride a bicycle in grade 3 or 4	no calculation of new variables required
	vaccination rate among children	diphtheria	the city public health department	non-aggregated data sets of the school entry examination:<br>number of vaccinations against diphtheria for every child in the data sets	calculation of a new variable *"child with at least 4 vaccinations against diphtheria"* (basic immunization)<br><br>calculation of the percentage *(children with at least 4 vaccinations*

				*against diphtheria/ children examined in the school entry examination) * 100*
	tick-borne encephalitis (TBE)	the city public health department	non-aggregated data sets of the school entry examination:<br>number of vaccinations against tick-borne encephalitis (TBE) for every child in the data sets	calculation of a new variable *"child with at least 3 vaccinations against tick-borne encephalitis (TBE)"* (basic immunization)<br><br>calculation of the percentage *(children with at least 3 vaccinations against tick-borne encephalitis (TBE)/ children examined in the school entry examination) * 100*

hepatitis B	the city public health department	non-aggregated data sets of the school entry examination: number of vaccinations against hepatitis B for every child in the data sets	calculation of a new variable *"child with at least 4 vaccinations against hepatitis B"* (basic immunization)<br><br>calculation of the percentage *(children with at least 4 vaccinations against hepatitis B/ children examined in the school entry examination) * 100*
hemophilus influenza type b (Hib)	the city public health department	non-aggregated data sets of the school entry examination: number of vaccinations against hemophilus influenza type b (Hib) for every child in the data sets	calculation of a new variable *"child with at least 4 vaccinations against hemophilus influenza type b (Hib)"* (basic immunization)

			calculation of the percentage *(children with at least 4 vaccinations against hemophilus influenza type b (Hib)/ children examined in the school entry examination) * 100*
measles	the city public health department	non-aggregated data sets of the school entry examination: number of vaccinations against measles for every child in the data sets	calculation of a new variable *"child with at least 2 vaccinations against measles"* (basic immunization)<br><br>calculation of the percentage *(children with at least 2 vaccinations*

			*against measles/ children examined in the school entry examination) * 100*
meningococci	the city public health department	non-aggregated data sets of the school entry examination:<br>number of vaccinations against meningococci for every child in the data sets	calculation of a new variable *"child with at least 1 vaccination against meningococci"* (basic immunization)<br><br>calculation of the percentage *(children with at least 1 vaccination against meningococci/ children examined in the school entry examination) * 100*

mumps	the city public health department	non-aggregated data sets of the school entry examination: number of vaccinations against mumps for every child in the data sets	calculation of a new variable *"child with at least 2 vaccinations against mumps"* (basic immunization)<br><br>calculation of the percentage *(children with at least 2 vaccinations against mumps/ children examined in the school entry examination) * 100*
pertussis (whooping cough)	the city public health department	non-aggregated data sets of the school entry examination: number of vaccinations against pertussis (whooping cough) for every child in the data sets	calculation of a new variable *"child with at least 4 vaccinations against pertussis (whooping cough)"* (basic immunization)

			calculation of the percentage *(children with at least 4 vaccinations against pertussis (whooping cough)/ children examined in the school entry examination) * 100*
pneumococci	the city public health department	non-aggregated data sets of the school entry examination:<br>number of vaccinations against pneumococci for every child in the data sets	Different calculations for the data sets since 2015 and until 2015 due to a change in the definition of basic immunization<br><br>since 2015:<br>calculation of a new variable *"child with at least 3*

			vaccinations against pneumococci" (basic immunization)<br><br>calculation of the percentage *(children with at least 3 vaccinations against pneumococci/ children examined in the school entry examination) * 100*<br><br><u>until 2015:</u> calculation of a new variable *"child with at least 4 vaccinations against pneumococci"* (basic immunization)

			calculation of the percentage *(children with at least 4 vaccinations against pneumococci/ children examined in the school entry examination) * 100*
poliomyelitis	the city public health department	non-aggregated data sets of the school entry examination:<br>number of vaccinations against poliomyelitis for every child in the data sets	calculation of a new variable *"child with at least 4 vaccinations against poliomyelitis"* (basic immunization)<br><br>calculation of the percentage *(children with at least 4 vaccinations against poliomyelitis/ children examined in*

			*the school entry examination) * 100*
rubella	the city public health department	non-aggregated data sets of the school entry examination:<br>number of vaccinations against rubella for every child in the data sets	calculation of a new variable *“child with at least 2 vaccinations against rubella”* (basic immunization)<br><br>calculation of the percentage *(children with at least 2 vaccinations against rubella/ children examined in the school entry examination) * 100*
tetanus	the city public health department	non-aggregated data sets of the school entry examination:	calculation of a new variable *“child with at least 4 vaccinations against*

		number of vaccinations against tetanus for every child in the data sets	*tetanus"* (basic immunization)<br><br>calculation of the percentage *(children with at least 4 vaccinations against tetanus/ children examined in the school entry examination) * 100*
varicella (chicken-pox)	the city public health department	non-aggregated data sets of the school entry examination:<br>number of vaccinations against varicella (chicken-pox) for every child in the data sets	calculation of a new variable *"child with at least 2 vaccinations against varicella"* (basic immunization)<br><br>calculation of the percentage *(children with at least 2 vaccinations*

				*against varicella (chicken-pox)/ children examined in the school entry examination) * 100*
utilization of health screenings in early childhood	% participated in health examinations in the age between 4 to 5 weeks to 1 year	the city public health department	non-aggregated data sets of the school entry examination: information whether a child participated in different health examinations for every child in the data sets	calculation of a new variable *"child that participated in all four health examinations in the age between 4 to 5 weeks to 1 year"*<br><br>calculation of the percentage *(children participated in all four health examinations in the age between 4 to 5 weeks to 1 year/ children examined in*

			*the school entry examination) * 100*
% participated in health examination in the age between 1 year 9 months to 2 years	the city public health department	non-aggregated data sets of the school entry examination: information whether a child participated in the health examination for the age group for every child in the data sets	calculation of a new variable *"child that participated in the health examination in the age between 1 year 9 months to 2 years"*<br><br>calculation of the percentage *(children participated in the health examination in the age between 1 year 9 months to 2 years/ children examined in the school entry examination) * 100*

% participated in health examination in the age between 2 years 10 months to 3 years	the city public health department	non-aggregated data sets of the school entry examination: information whether a child participated in the health examination for the age group for every child in the data sets	calculation of a new variable *"child that participated in the health examination in the age between 2 years 10 months to 3 years"*<br><br>calculation of the percentage *(children participated in the health examination in the age between 2 years 10 months to 3 years/ children examined in the school entry examination) * 100*

	% participated in health examination in the age between 3 years 10 months to 4 years	the city public health department	non-aggregated data sets of the school entry examination: information whether a child participated in the health examination for the age group for every child in the data sets	calculation of a new variable *"child that participated in the health examination in the age between 3 years 10 months to 4 years"*<br><br>calculation of the percentage *(children participated in the health examination in the age between 3 years 10 months to 4 years/ children examined in the school entry examination) * 100*

child weight	% of underweight children[19]	the city public health department	non-aggregated data sets of the school entry examination:<br>since 2013:<br>information on percentiles for every child in the data sets<br><br>until 2013:<br>information on weight (kg), size (cm), age (years) and gender for every child in the data sets	since 2013:<br>calculation of the percentage *(children below the 10th percentile according to Kromeyer-Hauschild / children examined in the school entry examination) * 100*<br><br>until 2013:<br>calculation of new variables “*size (m)*”, *“BMI (weight (kg)/ (size (m))²)”, “percentiles”, “child below the 10th percentile according to Kromeyer-*

[19] BMI below the 10th percentile according to Kromeyer-Hauschild (Kromeyer-Hauschild et al. Perzentile für den Body-mass-Index für das Kindes-und Jugendalter unter Heranziehung verschiedener deutscher Stichproben. Monatsschrift Kinderheilkunde. **2001**, 149(8), 807-818).

				Hauschild" using different threshold values per age and gender according to Kromeyer-Hauschild<br><br>calculation of the percentage *(children below the 10th percentile according to Kromeyer-Hauschild / children examined in the school entry examination) * 100*
	% of overweight children[20]	the city public health department	non-aggregated data sets of the school entry examination: <u>since 2013:</u>	<u>since 2013:</u> calculation of the percentage

[20] BMI above the 90th percentile according to Kromeyer-Hauschild (Kromeyer-Hauschild et al. Perzentile für den Body-mass-Index für das Kindes-und Jugendalter unter Heranziehung verschiedener deutscher Stichproben. Monatsschrift Kinderheilkunde. **2001**, 149(8), 807-818).

	information on percentiles for every child in the data sets	*(children above the 90th percentile according to Kromeyer-Hauschild / children examined in the school entry examination) * 100*
	<u>until 2013:</u><br>information on weight (kg), size (cm), age (years) and gender for every child in the data sets	<u>until 2013:</u><br>calculation of new variables “*size (m)*”, “*BMI (weight (kg)/ (size (m))^2)*”, “*percentiles*”, “*child above the 90th percentile according to Kromeyer-Hauschild*” using different threshold values per age and gender according

			to Kromeyer-Hauschild<br><br>calculation of the percentage *(children above the 90th percentile according to Kromeyer-Hauschild / children examined in the school entry examination) * 100*
% of obese children[21]	the city public health department	non-aggregated data sets of the school entry examination:<br>since 2013:<br>information on percentiles for every child in the data sets	since 2013:<br>calculation of the percentage *(children above the 97th percentile according to Kromeyer-Hauschild / children examined*

[21] BMI above the 97th percentile according to Kromeyer-Hauschild (Kromeyer-Hauschild et al. Perzentile für den Body-mass-Index für das Kindes-und Jugendalter unter Heranziehung verschiedener deutscher Stichproben. Monatsschrift Kinderheilkunde. **2001**, 149(8), 807-818).

			*in the school entry examination) * 100*
		<u>until 2013:</u><br>information on weight (kg), size (cm), age (years) and gender for every child in the data sets	<u>until 2013:</u><br>calculation of new variables “*size (m)*”, “*BMI (weight (kg)/ (size (m))^2)*”, “*percentiles*”, “*child above the 97th percentile according to Kromeyer-Hauschild*” using different threshold values per age and gender according to Kromeyer-Hauschild<br><br>calculation of the percentage

					*(children above the 97th percentile according to Kromeyer-Hauschild / children examined in the school entry examination) * 100*
	utilization of the early childhood intervention (ECI) service “Welcome to Life”	% of parents with newborns participating in early childhood intervention service „Welcome to Life“	the youth welfare office	non-aggregated data sets of the early childhood intervention service „Welcome to Life“	calculation of the percentage *(parents in the data set of the early childhood intervention service „Welcome to Life“/births) * 100*
			the local statistics office	aggregated register data of the local statistics office: absolute figures for births	
personal security	crimes	number of total crimes per 1.000 inhabitants	the local police department	aggregated police statistics: number of total crimes	calculation of the number of total crimes per 1.000 inhabitants *(number of total crimes * 1000) / main resident population*
			the local statistics office	aggregated register data of the local statistics office: absolute figures for the main resident population	

number of violent crimes per 1.000 inhabitants	the local police department<br>the local statistics office	aggregated police statistics: number of violent crimes<br>aggregated register data of the local statistics office: absolute figures for the main resident population	calculation of the number of violent crimes per 1.000 inhabitants<br>*(number of violent crimes * 1000) / main resident population*
number of motor-vehicle thefts per 1.000 inhabitants	the local police department<br>the local statistics office	aggregated police statistics: number of motor-vehicle thefts<br>aggregated register data of the local statistics office: absolute figures for the main resident population	calculation of the number of motor-vehicle thefts per 1.000 inhabitants<br>*(number of motor-vehicle thefts * 1000) / main resident population*
number of property damages per 1.000 inhabitants	the local police department<br>the local statistics office	aggregated police statistics: number of property damages<br>aggregated register data of the local statistics office:	calculation of the number of property damages per 1.000 inhabitants<br>*(number of property damages * 1000) /*

			absolute figures for the main resident population	*main resident population*
	number of domestic burglaries per 1.000 inhabitants	the local police department	aggregated police statistics: number of domestic burglaries	calculation of the number of domestic burglaries per 1.000 inhabitants *(number of domestic burglaries * 1000) / main resident population*
		the local statistics office	aggregated register data of the local statistics office: absolute figures for the main resident population	
drug-related crime	number of total drug-related crimes per 1.000 inhabitants	the local police department	aggregated police statistics: number of total drug-related crimes	calculation of the number of total drug-related crimes per 1.000 inhabitants *(number of total drug-related crimes * 1000) / main resident population*
		the local statistics office	aggregated register data of the local statistics office: absolute figures for the main resident population	

	number of convictions due to illegal drug trafficking per 1.000 inhabitants	the local police department	aggregated police statistics: number of convictions due to illegal drug trafficking	calculation of the number of convictions due to illegal drug trafficking per 1.000 inhabitants *(number of convictions due to illegal drug trafficking * 1000) / main resident population*
		the local statistics office	aggregated register data of the local statistics office: absolute figures for the main resident population	
road accidents	number of road accidents per 1.000 inhabitants	the local police department	non-aggregated data sets of road accidents	calculation of the proportion *(number of road accidents * 1000) / main resident population*
		the local statistics office	aggregated register data of the local statistics office: absolute figures for the main resident population	
	number of road accidents involving cyclists or pedestrians per 1.000 inhabitants	the local police department	non-aggregated data sets of road accidents:	calculation of a new variable: *"road accidents involving*

	the local statistics office	information on the involvement of different road users for every road accident in the data sets<br><br>aggregated register data of the local statistics office: absolute figures for the main resident population	*cyclists or pedestrians* "<br><br>calculation of the proportion<br>*(road accidents involving cyclists or pedestrians * 1000) / main resident population*
number of road accidents with personal injury per 1.000 inhabitants	the local police department<br><br>the local statistics office	non-aggregated data sets of road accidents: information on the number of deaths, serious injuries and minor injuries for every road accident in the data sets<br><br>aggregated register data of the local statistics office: absolute figures for the main resident population	calculation of a new variable: "*accident with personal injury* "<br><br>calculation of the proportion<br>*(number of road accidents with personal injury * 1000) / main resident population*

Table S2 "The parking Lot": Indicators desired by participants for which no data were available and could therefore not be incorporated in the field-ready version of the neighborhood barometer

dimension	categories	indicators
population structure	disability	persons with disabilities
	care needs	persons with care needs
population development	natural population development	life expectancy
		age-standardized mortality rate
		causes of mortality
material wellbeing (economics)	employment / unemployment	average net household income
		persons in marginal employment and „top-up" employees
	recipients of social benefits	utilization of the "Education and Participation Package"[22]
	debt	rent arrears rate
		per capita debt
education	school absenteeism	students with school anxiety (anxiety-induced absenteeism)
		students playing truant (absence actively decided by the student)
		students who are held back from lessons (e.g. by parents)
	training	young people without training
		trainees
	secondary school	young people without a lower secondary school leaving certificate
family and upbringing	Family Pass	utilization of the Family Pass[23]

[22] The German government offers financial support for the education and participation of children of families receiving social security or unemployment benefits. Costs for school supplies, excursions or participation in leisure activities are exemplary for the governmental support.

[23] An offer for all families and single parents living in the city of Mannheim including a voucher booklet with authorization card (e.g., vouchers for indoor swimming pools, the theatre or the city parks)

	kindergarten	children who attended kindergarten < two years
	youth welfare	utilization of 'Early support centers for parent and child support'
		youth work
child health	factors influencing health status	children with a birth weight below 2500g
		breastfed children
		physical activity behavior
		dietary habits
		maternal smoking during pregnancy
		use of digital media
		exposure to second-hand smoke
	health status	children with allergies
		children with chronic diseases
		children with infectious diseases
		subjective health status
		oral health
		children with mental illness
	experience of emotional distress	children experiencing loneliness
		children who experience bullying
personal security	perception of security	perception of security
adolescent health	factors influencing health status	alcohol consumption
		physical activity behavior
		dietary habits
		weight (underweight/ overweight/ obesity)
		exposure to second-hand smoke
		smoking behavior
		adolescents able to swim
		adolescents able to ride a bicycle safely
	health status	adolescents with allergies
		adolescents with chronic diseases
		adolescents with infectious diseases
		subjective health status

		life satisfaction
		oral health
		immunization coverage
		young people with mental illness
	experience of emotional distress	young people experiencing loneliness
		young people who experience bullying
		abortions
adult health	factors influencing health status	alcohol consumption
		physical activity behavior
		dietary habits
		weight (underweight/ overweight/ obesity)
		exposure to second-hand smoke
		smoking behavior
		adults able to swim
		adults who able to a bicycle safely
	health status	adults with allergies
		adults with chronic diseases
		adults with infectious diseases
		subjective health status
		life satisfaction
		oral health
		immunization coverage
		adults with mental illness
	experience of emotional distress	adults experiencing loneliness
		adults who experience bullying
		abortions
senior health	factors influencing health status	alcohol consumption
		physical activity behavior
		dietary habits
		weight (underweight/ overweight/ obesity)
		exposure to second-hand smoke
		smoking behavior
		seniors able to swim
		seniors able to ride a bicycle safely

	health status	seniors with allergies
		seniors with chronic diseases
		seniors with infectious diseases
		subjective health status
		life satisfaction
		oral health
		immunization coverage
		seniors with mental illness
	experience of emotional distress	seniors experiencing loneliness
Structural framework conditions of healthcare	healthcare	density of pharmacies
		density of medical specialists
		density of general practitioners
		density of pediatricians
		density of dentists
living conditions and surroundings	land use	total share of built-up areas
		total share of green areas
		public parking space in the neighborhood
	quality of living	perceived accessibility in the neighborhood
		perceived heat stress
		perceived air quality
		perceived noise pollution
		perceived cleanliness
		frequency of use of leisure programs in the neighborhood
		satisfaction with leisure programs
		perception of meeting places in the neighborhood
	attitudes towards the neighborhood	satisfaction with the neighborhood
		connectedness to the neighborhood
	housing stock	share of private property
		share of social housing
		barrier-free housing

		rental index
		living space per capita
		housing quality (e.g. mould)
		housing vacancies (vacant dwellings)
mobility	radius of action	children's radius of action
		adolescent’s radius of action
participation and involvement	voluntary work	number of associations
		membership in associations
	networks	neighborhood cohesion
		existence of social networks
	electoral turnout	electoral turnout in federal elections
		electoral turnout in state elections
